# Teaching sustainability through disaster simulation: A framework for planetary health education

**DOI:** 10.1186/s41077-026-00433-y

**Published:** 2026-03-25

**Authors:** Nicole Petsas Blodgett, Valerie K. Sabol, AnnMarie Lee Walton

**Affiliations:** Duke University School of Nursing, DUMC 3322, 307 Trent Drive, Durham, North Carolina 27710 USA

**Keywords:** Adaptation, Climate change, Disaster, Mitigation, Simulation, Sustainability

## Abstract

**Background:**

Climate‑driven disasters are increasing in frequency and severity, creating prolonged recovery periods and widening existing health inequities. As extreme weather events intensify, healthcare professionals must be prepared to mitigate risks, adapt to evolving conditions, and understand how environmental degradation influences population health. Simulation‑based education provides a safe, flexible platform for introducing these concepts while fostering core competencies in disaster preparedness.

**Approaches:**

This article outlines strategies for integrating planetary health and sustainability principles into climate‑driven disaster simulations across preparedness, response, and recovery phases. Approaches include using low‑waste modalities such as tabletop, hybrid, and digital simulations; repurposing expired non‑sterile supplies; and designing scenarios that explicitly incorporate resource stewardship. We distinguish between sustainability as curricular content (what learners are taught) and sustainability as operational practice (how simulations are conducted), arguing that both must be addressed to reduce the environmental footprint of training while advancing climate fluency. Embedding sustainability objectives into learning outcomes, debriefings, and evaluation metrics enables learners to examine both their clinical decision‑making and the environmental implications of their actions. The article also highlights opportunities to incorporate climate and sustainability concepts across undergraduate and graduate curricula. These strategies promote systems thinking, strengthen interprofessional collaboration, and support the development of clinicians who understand the links between climate change, health disparities, and responsible use of healthcare resources.

**Conclusion:**

By integrating sustainability, mitigation, and adaptation principles into disaster simulations, educators can prepare future healthcare professionals to respond effectively to climate‑driven crises while reducing the environmental impact of training itself. This intentional approach strengthens disaster readiness and cultivates a culture of environmental responsibility across health professional education This dual approach reframes disaster simulation as both clinical preparation and climate action within health professions education.

## Background

In 2024, the United States experienced 27 separate weather or climate-driven disasters. Each of these resulted in at least $1 billion dollars in damages [1]. Climate-driven disasters occur as a result of climate change. The National Academies reported that recent studies in attribution science show that climate change is causing an increase in the frequency and/or severity of tropical storms, heavy rainfall, and extreme temperatures. The intensification of these and other more extreme weather-climate events are intersecting with areas experiencing high levels of health disparities, social vulnerabilities, and increased exposure due to population growth in hazard-prone areas. This convergence is resulting in prolonged and overlapping periods of disaster recovery [2]. Healthcare professionals must be prepared for these prolonged periods of disaster recovery and their resultant health effects.

Planetary health is an emerging field that recognizes the interdependence between human health and the state of natural systems. It emphasizes that the health of human populations is inseparable from the health of the planet and its ecosystems. Planetary health concepts must be taught to nurses, physicians, and other health professionals [3–5]. However, competencies, content, and teaching methods in planetary health education are heterogeneous, and innovative methods are needed [6]. Training healthcare professionals to mitigate and adapt to climate-driven disasters by reducing waste, identifying risks, preserving health, and preventing ongoing complications is of the utmost importance. Climate-driven disaster simulation curricula should integrate planetary health principles explicitly, linking local disaster preparedness with global environmental health outcomes. Students should engage in reflective debriefing that includes discussions about how climate change drives disaster frequency and severity, fostering both technical competence and environmental awareness.

A core component of planetary health is addressing climate change, which is human-driven alterations in climate patterns caused by increased greenhouse gas (GHG) emissions [7]. Climate action encompasses two strategies: mitigation, which reduces or prevents emissions, and adaptation, which minimizes health and environmental impacts, particularly for vulnerable populations [8]. Training healthcare professionals to mitigate and adapt to climate-driven disasters is essential for reducing waste, identifying risks, and preventing complications. The increasing frequency, intensity, and duration of extreme weather events present complex clinical, ethical, and resource-related challenges for health systems worldwide [8]. Accordingly, climate disaster simulations should explicitly integrate planetary health principles, linking local preparedness to global environmental outcomes and fostering reflection on how climate change drives disaster frequency and severity. To date, however, disaster simulations have largely focused on clinical competence and operational efficiency without explicitly addressing the environmental impact of training itself or integrating sustainability principles into learning objectives. This article argues that disaster simulation offers a powerful yet underutilized opportunity to integrate planetary health, mitigation, and adaptation principles into health professions education. We propose a structured approach to designing sustainable climate-informed disaster simulations that advance both disaster preparedness and environmental stewardship. It has long been recognized that simulation-based learning provides a safe yet realistic environment to practice disaster responses while grappling with contextual pressures like health inequities, resource scarcity, and strained healthcare infrastructures [9–11].

### Integrating sustainability mindset into disaster preparedness simulations

Disaster preparedness training is essential for equipping healthcare professionals with the knowledge, skills, and confidence to respond to increasingly frequent and severe events such as hurricanes, wildfires, floods, and pandemics. These simulations provide invaluable opportunities for practice under controlled, high-pressure conditions and are critical for ensuring a rapid, coordinated response when real-world disasters occur.

However, while the intent behind these training efforts is to improve health system resilience and save lives, there are unintended consequences that must be acknowledged. Large-scale disaster simulations often require significant amounts of single-use equipment, consumables, and energy [12]. Therefore, disaster simulations generate substantial waste, much of which ends up in landfills or contributes to GHG emissions through incineration. Ironically, in striving to protect communities and prepare for future crises, healthcare education programs may inadvertently contribute to the very environmental degradation that fuels climate-driven disasters.

Healthcare waste has been well-described [13]. Less has been written about healthcare education waste. Little is known about low-cost, sustainable solutions that teach mitigation of climate change through individual and practice-based solutions while also training healthcare professions (HCP) educators to teach about climate-driven disasters. What is known about sustainability education is that when students were educated about resource stewardship and sustainability in clinical simulation, students perceived application of sustainability during clinical simulation and clinical practice improved [14]. Developing sustainable practices requires collaboration not only with academic administrators but with hospital leaders and others who make decisions about recycling [15]. Lifecycle analyses of simulation equipment to identify upstream carbon emissions and opportunities for circular economy approaches. For example, reusing expired clinical supplies, redesigning simulation kits to avoid unnecessary materials, and working with manufacturers to implement take-back programs for single-use plastics can dramatically reduce waste. Additionally, students can debrief about choice, use, and outcomes of supplies in the healthcare setting. These approaches elevate simulation from skill acquisition to leadership preparation, equipping future clinicians to balance clinical excellence with environmental responsibility. The purpose of this article is to describe how HCP educators can create climate-driven disaster healthcare simulations that include critically important content about mitigation and adaptation leadership.

### Planning and development

Simulation is the ideal approach to learn about climate-driven disasters impact on population health. It is vital to design the experience around using Healthcare Simulation Standards of Best Practices [16], paying particular attention to simulation design and learning objectives. Consideration of use of simulation to prepare healthcare students at several different timeframes (or phases) of the disaster: (1) Preparedness, (2) Response and (3) Recovery (Table 1). Using the timeframes of a disaster will help identify learners’ needs assessment. Embedding sustainability explicitly into learning objectives ensures that learners reflect not only on clinical decision-making but also on environmental implications of supply use, transportation, energy consumption, and waste management.

**Table 1:** Phases of disaster simulations with focus on climate friendly solutions

Phases	Intrinsic Factors	Extrinsic Factors	Potential Simulations with a Planetary Health Focus
Preparedness – reduce risk before the disaster	Medications Ambulation Age Income Family Preparedness	Environment Location Climate Insurance/Access to Care Community Preparedness	Table-top simulation. Create case study. Video of monologue of community members. Community Assessment
Response - Managing the Immediate Disaster	Immediate safety Triage Medical needs	Water Food Shelter	Triage simulation Communication Simulation Dry-run/walk through of action plan
Recovery - preserving health after the disaster	Resilience Income Agility	Access to medications Access to healthcare provider Access to assistance Water Food Shelter	Role play Provider visit (post-event, etc.) Interview patient as a healthcare provider

For example, in the preparedness phase, learners could complete a community assessment using identified intrinsic and extrinsic factors of the population. Then, learners could develop community preparation plans for various types of environmental emergencies. This approach can align core competencies in disaster aid to the learning objectives of the simulation (Table 2) [17]. When planning disaster-based simulations that are linked to competencies, careful consideration of low-cost, sustainable solutions should be considered.

**Table 2:** Aligning disaster simulation objectives with competencies: An exemplar

Disaster Competency	Course Objective	Simulation Objective	Simulation Design to meet objectives
Undergraduate Domain 1: Preparation and Planning I.1.1 Maintains a general personal, family and professional preparedness plan (International Council of Nurses, 2019)	Apply theoretical knowledge and practical skills to demonstrate competency in disaster preparedness and response.	Create a preparedness plan for your personal needs, family needs, and professional.	Students work in table-top simulations to co-create personal and professional preparedness plans
Graduate Domain 2: Communication II.2.3 Collaborates with disaster leadership team(s) to develop event-specific media messages (International Council of Nurses, 2019)	Manage population health using evidence-based interventions.	Create social media posts using evidence-based interventions following a disaster.	Watch a recorded newscast of a recent disaster event (as a case study). Students will work in groups to created social media posts promoting evidence-based interventions (food, water, masking, etc.)

The benefit of simulation is the learner has the opportunity for active experimentation and reflection on action [18]. This reflection can occur during the simulation and after the simulation. This may be incorporated in the simulation design by including post-simulation reflection on the environmental impact of the event. Extending learning beyond the simulation for further learning about environmental impact of healthcare is in alignment with simulation facilitation best practices [19].

#### Entry-level healthcare education

With a broader planetary health and sustainability mindset, healthcare education programs provide an ideal entry point for embedding climate-driven disaster preparedness. While traditional disaster simulations have emphasized acute technical skills, integrating planetary health and sustainability principles into tabletop simulations creates opportunities for learners to link local disaster responses to global climate disruption. For example, when healthcare students role-play hurricane-response scenarios, facilitators can include prompts that highlight how climate change has intensified storm frequency, severity, and duration by using data from national reports [9–11]. This layered approach provides higher-order critical thinking and situates technical decision-making within real-world ecological and social contexts.

#### Graduate education

Graduate-level healthcare and interprofessional programs require learners to move beyond individual skill-building toward systems-level thinking, leadership, and quality improvement (QI). Disaster preparedness simulations at this level can explicitly integrate sustainability audits, supply chain analysis, and systems resilience planning, preparing future care providers to balance clinical excellence with environmental responsibility [4, 6]. Graduate QI projects, for example, might include piloting reusable or modular equipment kits for disaster training or conducting life-cycle analyses of single-use simulation materials to measure their downstream impacts on GHG emissions and local communities [14]. These QI projects not only strengthen critical evaluation skills but also provide learners with tangible strategies to reduce the unintended environmental consequences of healthcare education, skills they can carry forward into professional practice environments.

Finally, the use of validated simulation evaluation tools, such as the Simulation Effectiveness Tool - Modified, can provide evidence of simulation effectiveness for disaster-based simulations and is essential [16, 20]. Incorporating climate-mitigation skill acquisition and learners’ perceptions of sustainability awareness can be incorporated into the debriefings. We recommend expanding evaluation to include sustainability-related outcomes, such as learner awareness of resource stewardship, ability to articulate mitigation and adaptation strategies, and application of systems thinking to environmental impact.

### Sustainable teaching innovations

#### Disaster-based tabletop simulations

Tabletop simulation activities have been used to enhance healthcare students’ capacity to respond to extreme weather events and students reported enhanced critical thinking, decision making, collaboration, teamwork, preparedness, and adaptability in crisis situations [21]. In these tabletop simulation exercises, students were given unfolding situations to work with and to learn how healthcare priorities may change over time and in response to the needs of the people in the scenario with little to no impact on the environment [21]. Unlike large scale disaster simulations, which often demand significant time, costly resources, and generate significant waste, tabletop simulation activities have demonstrated effectiveness in strengthening students’ adaptability, teamwork, and crisis decision-making [21–23]. A tabletop approach also aligns with the imperatives of planetary health through reduced use of complex or expensive equipment and supplies. For example, mannikins can be replaced by posters and instead of real equipment, learners apply printed images (oxygen mask, tourniquet, intravenous fluids, etc.) onto the patient [24]. Programs can also mitigate the environmental impacts by repurposing expired but clean supplies for non-sterile training, preserving realism while reducing landfill waste and modeling resource stewardship [14]. This combination of low-cost, high-impact training not only builds disaster readiness but also embeds sustainability as a professional norm from the earliest stages of healthcare education.

#### Disaster-based hybrid and digital simulation

Simulation educators have also demonstrated that incorporating hybrid and digital simulation can maintain educational fidelity while achieving significant costs and waste reductions. In Australia, for instance, sustainability principles were embedded into graduate disaster simulations, requiring learners to evaluate both the effectiveness of their clinical decision-making and the environmental impact of their chosen responses [15]. Such approaches expand traditional definitions of preparedness by linking disaster response competencies to broader planetary health objectives, and sustainability becomes both a design principle and learning objective.

By aligning graduate curricula with planetary health and interprofessional collaboration standards, educators can ensure that advanced learners graduate with the ability to lead disaster response efforts that are both clinically sound and environmentally sustainable.

### Lessons learned and recommendations

By integrating a sustainability mindset into disaster preparedness education, educators can strike a balance between effective skill-building and responsible resource use. Recognizing that our resources are not limitless, simulation teams can intentionally design scenarios that maximize educational impact while minimizing environmental harm. For example, expensive, non-recyclable supplies can be reserved for critical moments that require a high level of realism in a simulation, while lower level of realism or digital tools can be used for initial training and tabletop exercises. Expired but clean clinical supplies can be repurposed for non-sterile training purposes, diverting them from waste streams while preserving the realism necessary for disaster training [14, 25]. Innovative practices such as incorporating virtual reality simulation, case-based learning, or hybrid tabletop simulation exercise can also reduce environmental costs while maintaining learner engagement [15] (Table 3). Simulation offers a unique opportunity to teach sustainable practices explicitly. For example, learners can critically assess supply choices during a disaster drill, compare the carbon footprint of different response strategies, or calculate potential waste through reuse and recycling programs [11]. In this way, the simulation environment itself becomes a living laboratory for sustainability education.

**Table 3:** Practical steps to integrate sustainability into disaster preparedness training

Sustainability into Disaster Preparedness Simulation
Developing an inventory of reusable materials specifically designated for disaster simulations
Implementing clear waste segregation and recycling systems within the simulation space
Partnering with suppliers to establish take-back programs for plastics and packaging.
Tracking and reporting the environmental footprint of simulations alongside educational outcomes.
Embedding sustainability objectives into simulation design and evaluation metrics.

Some may argue that sustainability is a secondary concern during disaster training or actual disaster response; however, given the increasing frequency of climate-driven events, repeated high-waste training models may exacerbate long-term environmental strain. A more integrated approach acknowledges that disaster preparedness and environmental stewardship are not competing priorities but mutually reinforcing responsibilities. Climate-driven disasters are occurring with greater frequency, intensity and duration, which means more supplies and more training will be required, further straining already limited resources and compound GHG emissions [1, 9, 10]. Even the most carefully planned simulations still generate significant waste, reminding us that resource stewardship is not a luxury, but a necessity if we are to prepare for future healthcare professional responsibly [11, 14].

This approach also reframes the learning experience itself. During debriefings, facilitators can guide students in reflecting not only their disaster response performance but also on the environmental implications of the training. Discussions can explore questions such as: How does healthcare contribute to climate change and disaster frequency? How can we prepare for emergencies while being good stewards of our planet’s resources? These reflections link local simulation practices to the global planetary health context, fostering a deeper sense of responsibility among learners. Emerging evidence suggests that such reflective debriefings enhance both clinical competence and long-term retention of sustainability principles, reinforcing the dual goals of health system resilience and planetary health stewardship [4, 6].

Fostering a sustainability mindset requires leadership commitment and ongoing education of health professions colleagues. This critical reframing will position simulation centers as laboratories for climate-responsive healthcare education. Simulation center leaders must engage faculty, staff, and students through workshops, green teams, and incentive programs to maintain momentum. Institutional policies that integrate sustainability metrics into simulation accreditation or evaluation process may further accelerate adoption of environmentally responsible practices. More information about the effectiveness of these strategies needs to be addressed.

## Conclusion

Creating a sustainable, disaster-based healthcare simulation is possible. HCP educators can design disaster simulations with low-cost, sustainable solutions to training learners to be responsive to the current environment. A sustainability mindset transforms simulation centers into models of environmental responsibility and operational excellence. Through intentional design, efficient resource management, and collaborative culture, simulation centers can play a vital role in preparing future healthcare professionals to deliver care that is both high quality and environmentally sustainable. In doing so, simulation centers can not only strengthen disaster preparedness capacity but also contribute to the broader climate action agenda in health professional education. Integrating planetary health into disaster simulation ensures that future clinicians are prepared not only to respond to crises but also to anticipate, mitigate, and manage their impacts in ways that support long-term environmental sustainability and resilient health systems.

## Data Availability

No datasets were generated or analysed during the current study.
